# Machine learning-based CT radiomics approach for predicting WHO/ISUP nuclear grade of clear cell renal cell carcinoma: an exploratory and comparative study

**DOI:** 10.1186/s13244-021-01107-1

**Published:** 2021-11-20

**Authors:** Yingjie Xv, Fajin Lv, Haoming Guo, Xiang Zhou, Hao Tan, Mingzhao Xiao, Yineng Zheng

**Affiliations:** 1 Department of Radiology, The First Affiliated Hospital of Chongqing Medical University, No. 1 Youyi Road, Chongqing, 400016 Yuzhong China; 2 Department of Urology, The First Affiliated Hospital of Chongqing Medical University, No. 1 Youyi Road, Chongqing, 400016 Yuzhong China

**Keywords:** Machine learning, Tomography (X-ray computed), Clear cell renal cell carcinoma, WHO/ISUP grading, Prediction model

## Abstract

**Purpose:**

To investigate the predictive performance of machine learning-based CT radiomics for differentiating between low- and high-nuclear grade of clear cell renal cell carcinomas (CCRCCs).

**Methods:**

This retrospective study enrolled 406 patients with pathologically confirmed low- and high-nuclear grade of CCRCCs according to the WHO/ISUP grading system, which were divided into the training and testing cohorts. Radiomics features were extracted from nephrographic-phase CT images using PyRadiomics. A support vector machine (SVM) combined with three feature selection algorithms such as least absolute shrinkage and selection operator (LASSO), recursive feature elimination (RFE), and ReliefF was performed to determine the most suitable classification model, respectively. Clinicoradiological, radiomics, and combined models were constructed using the radiological and clinical characteristics with significant differences between the groups, selected radiomics features, and a combination of both, respectively. Model performance was evaluated by receiver operating characteristic (ROC) curve, calibration curve, and decision curve analyses.

**Results:**

SVM-ReliefF algorithm outperformed SVM-LASSO and SVM-RFE in distinguishing low- from high-grade CCRCCs. The combined model showed better prediction performance than the clinicoradiological and radiomics models (*p* < 0.05, DeLong test), which achieved the highest efficacy, with an area under the ROC curve (AUC) value of 0.887 (95% confidence interval [CI] 0.798–0.952), 0.859 (95% CI 0.748–0.935), and 0.828 (95% CI 0.731–0.929) in the training, validation, and testing cohorts, respectively. The calibration and decision curves also indicated the favorable performance of the combined model.

**Conclusion:**

A combined model incorporating the radiomics features and clinicoradiological characteristics can better predict the WHO/ISUP nuclear grade of CCRCC preoperatively, thus providing effective and noninvasive assessment.

**Supplementary Information:**

The online version contains supplementary material available at 10.1186/s13244-021-01107-1.

## Key points

- Nephrographic-phase CT radiomics is valuable in predicting the WHO/ISUP nuclear grade of CCRCC.
- Machine learning can noninvasively predict the WHO/ISUP nuclear grade of CCRCC.
- CT radiomics integrated with clinicoradiological parameters can facilitate differentiating between low- and high-grade CCRCCs with improved diagnostic efficacy.

## Introduction

Renal cell carcinoma accounts for 5% and 3% of all diagnosed cancers in men and women, respectively, and clear cell renal cell carcinoma (CCRCC) represents the most common subtype (∼ 80%) [1–3]. With a relatively poor prognosis, there is great interest in the field for improving diagnostic accuracy in order to start antineoplastic protocols at the early stage of CCRCC [4], because its biological aggressiveness significantly affects the prognosis. The pathological nuclear grade is an independent prognostic factor for CCRCC [5, 6]. Although the four-tiered Fuhrman grading system (FGS) for the pathological classification of CCRCC is widely used before, the 2016 World Health Organization/International Society of Urological Pathology (WHO/ISUP) grading system has achieved widespread usage and has now replaced the FGS globally [7, 8]. This system can be simplified as two-tiered classification combining grade I and II as low-grade and grade III and IV as high-grade. Moreover, low-grade cancers are generally considered less aggressive than high-grade ones [9]. The two-tiered classification has been verified to predict cancer-specific mortality and guide clinical practice in the same way as four-tiered systems, while it can reduce inter-observer variability and promote clinical practice [10, 11].

Percutaneous biopsy is a common method that can identify the pathology of the lesions, but it may be controversial because of invasive operation and sampling bias and even result in the increased risk of complications [12, 13]. Moreover, tumor heterogeneity refers to the existence of different subpopulations of cells, which can show distinct genotypes and divergent biological behaviors in different parts of a tumor. Thus, a noninvasive approach that can provide more information of lesions without the spatial and temporal restriction in tissue sampling is urgently needed, because it is too hard to biopsy each part of an entire tumor [14].

Despite its status as a routine noninvasive method to detect CCRCC, the routine computed tomography (CT) has the limitative power to differentiate renal cancer histologic grade with high consistency and accuracy [15]. Since resecting radiographically suspicious CCRCC without a tissue diagnosis is recommended, and this may lead to overtreatment in patients with low-grade CCRCC [16, 17], an exploration of the noninvasively preoperative differentiating between low- and high-nuclear grade of CCRCCs is urgent. Radiomics analysis enables the measurement of repetitive texture patterns at the voxel or pixel levels of medical images that are beyond the identification of the naked eye [18–20]. Previous investigations have shown that CT-based radiomics analysis performed efficiently in differentiating between low- and high-grade CCRCCs [21–24]. It might be a promising noninvasive assessment for predicting the nuclear grade of CCRCC. To our knowledge, most studies only constructed machine learning (ML) models using radiomics features extracted from CT images rather than a comprehensive model combined with those and clinicoradiological information. Furthermore, no previous studies have evaluated the performance of nephrographic-phase (NP) CT radiomics analysis for predicting the nuclear grade of CCRCC. Therefore, this study aims to investigate if radiomics features extracted from NP CT images combined with clinicoradiological characteristics may have potential in preoperatively differentiating the WHO/ISUP nuclear grade of CCRCC.

## Materials and methods

### Patient cohort

**Fig. 1:**
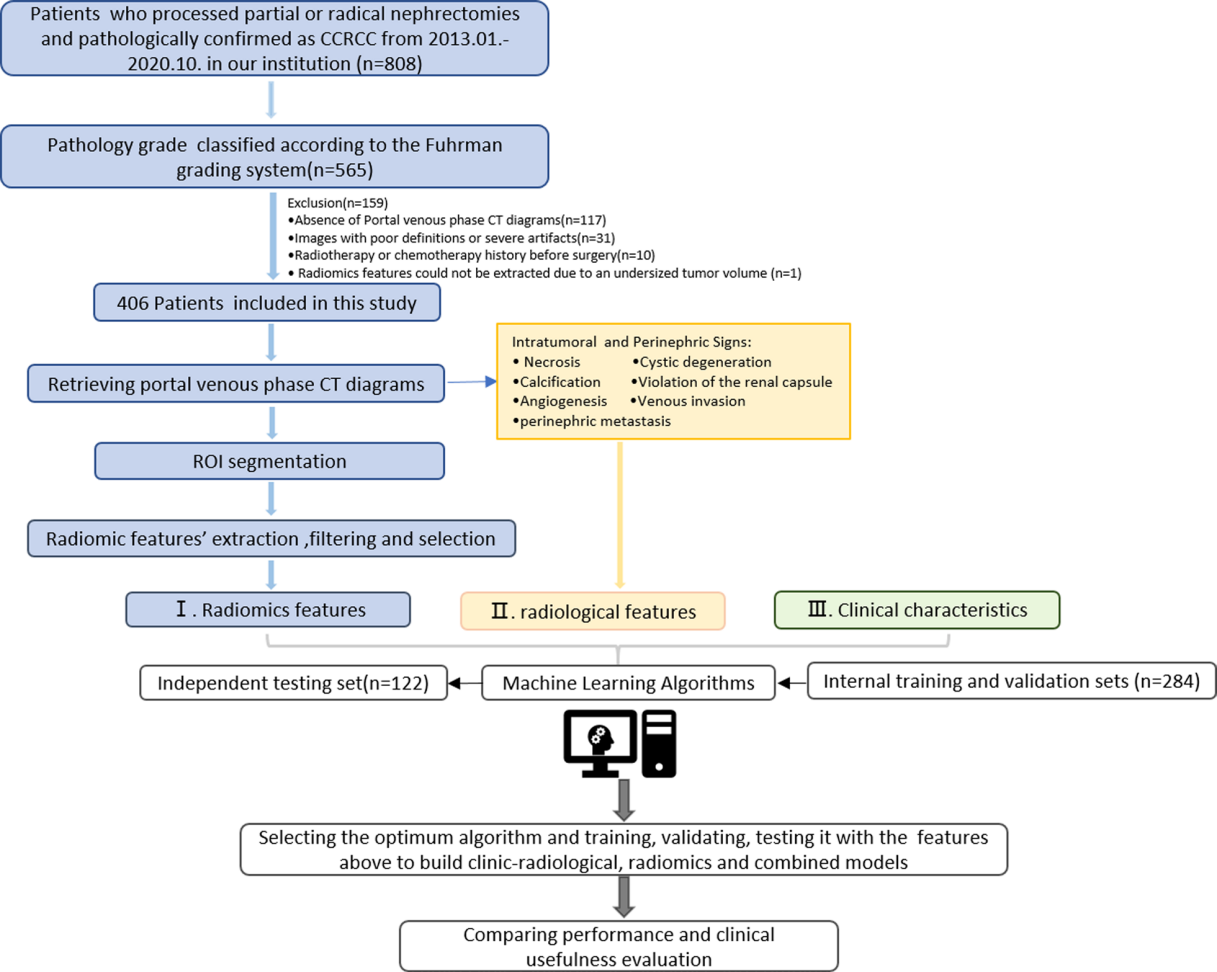
Flowchart of the procedures for this study (*CCRCC* clear cell renal cell carcinoma, *CT* computed tomography, *ROI* region of interest)

This retrospective study was approved by the Institutional Review Board of the First Affiliated Hospital of Chongqing Medical University, and the requirement for the acquisition of informed consent from patients was waived. The initial query yielded a target population of 808 patients with pathologically confirmed CCRCC who underwent partial or radical nephrectomy between January 2013 and October 2020 in our institution. Finally, a total of 406 patients with 330 low-grade and 76 high-grade CCRCCs were included in this study based on the following exclusion criteria: (1) pathology grade that was not classified according to the WHO/ISUP grading system (*n* = 243); (2) absence of NP CT images (*n* = 117); (3) images with poor definition or severe artifacts (*n* = 31); (4) a history of radiotherapy or chemotherapy before surgery (*n* = 10); and (5) radiomics features could not be extracted due to an undersized tumor volume (*n* = 1). The flowchart of this study is presented in Fig. 1. Moreover, the synthetic minority oversampling technique was used to increase the cases of high-grade CCRCC by oversampling for data balance [25].

### Nuclear grade and clinical characteristics

Two independent histopathological specialists re-evaluated each CCRCC sample regarding nuclear grade based on the criteria of the 2016 WHO/ISUP classification [8]. Discordant reports were resolved by a third senior histopathologist. We exhibit four hematoxylin–eosin staining slides with different magnifications from four patients with WHO/ISUP grading I–IV CCRCCs (Additional file 1: Figure S1). Data on the clinical characteristics that were presumed potentially grading-correlated (age, sex, body mass index [BMI], smoking history, hypertension history, diabetes history, tumor location, resection surgical procedure, etc.) and were extracted from the electronic medical record system of our institution.

### CT acquisition

All patients underwent a routine preoperative abdominal CT scan performed on a GE Discovery 750 HD (GE Healthcare, Milwaukee, WI) multidetector scanner. The parameters for CT imaging were as follows: tube voltage, 120–140 kV; tube current, 220–300 mAs; detector collimation, 0.625×64 mm; matrix, 512 × 512; slice thickness, 5 mm. All patients were injected with nonionic intravenous contrast agent, via the antecubital vein with mechanical power injector, according to their weight (1 mL/kg body weight, with a maximum of 150 mL). Phase and delay time were as follows: Phase 1, unenhanced; Phase 2, postcontrast corticomedullary phase (CMP): 25–28 s after contrast agent was administrated; Phase 3, postcontrast nephrographic phase (NP): 65–70 s after contrast agent was administrated; and Phase 4, postcontrast excretory phase [26].

### Image analysis

The semantic annotations of CT images and the corresponding diagnostic criteria were as follows: (a) tumor size, defined as the maximum diameter on transverse images; (b) intratumoral necrosis, defined as the non-enhanced fluid region of the tumor, which was greater than 50% of the tumor [27]; (c) cystic degeneration, defined as target lesion showing uniform water density and signal intensity, but no enhancement on enhancement examination [28]; (d) intratumoral calcification, interpreted as obvious dense shadows in the parenchyma that were speckled, lined, or shell-shaped; (e) violation of the renal capsule, interpreted as abnormal lesion violating the margin of the renal capsule; (f) intratumoral angiogenesis, defined as vascular enhancement observed in the parenchyma of the cortical stage tumor [27, 29]; (g) venous invasion, interpreted as radiological characteristics of tumor thrombosis in the renal vein and inferior vena cava [27]; (h) perinephric metastasis, defined as perinephric invasion phenomenon on CT images; and (i) distant metastasis, considered as metastasis in the lung, liver, bone, brain, or other organs via the blood or lymphatics. In our study, two radiologists with 10 or more years of experience in renal imaging who were blinded to histopathological results independently identified and evaluated these characteristics. Any discrepancy was resolved by reaching a consensus via discussion, and the results agreed on were used for further analysis.

### Tumor segmentation

**Fig. 2:**
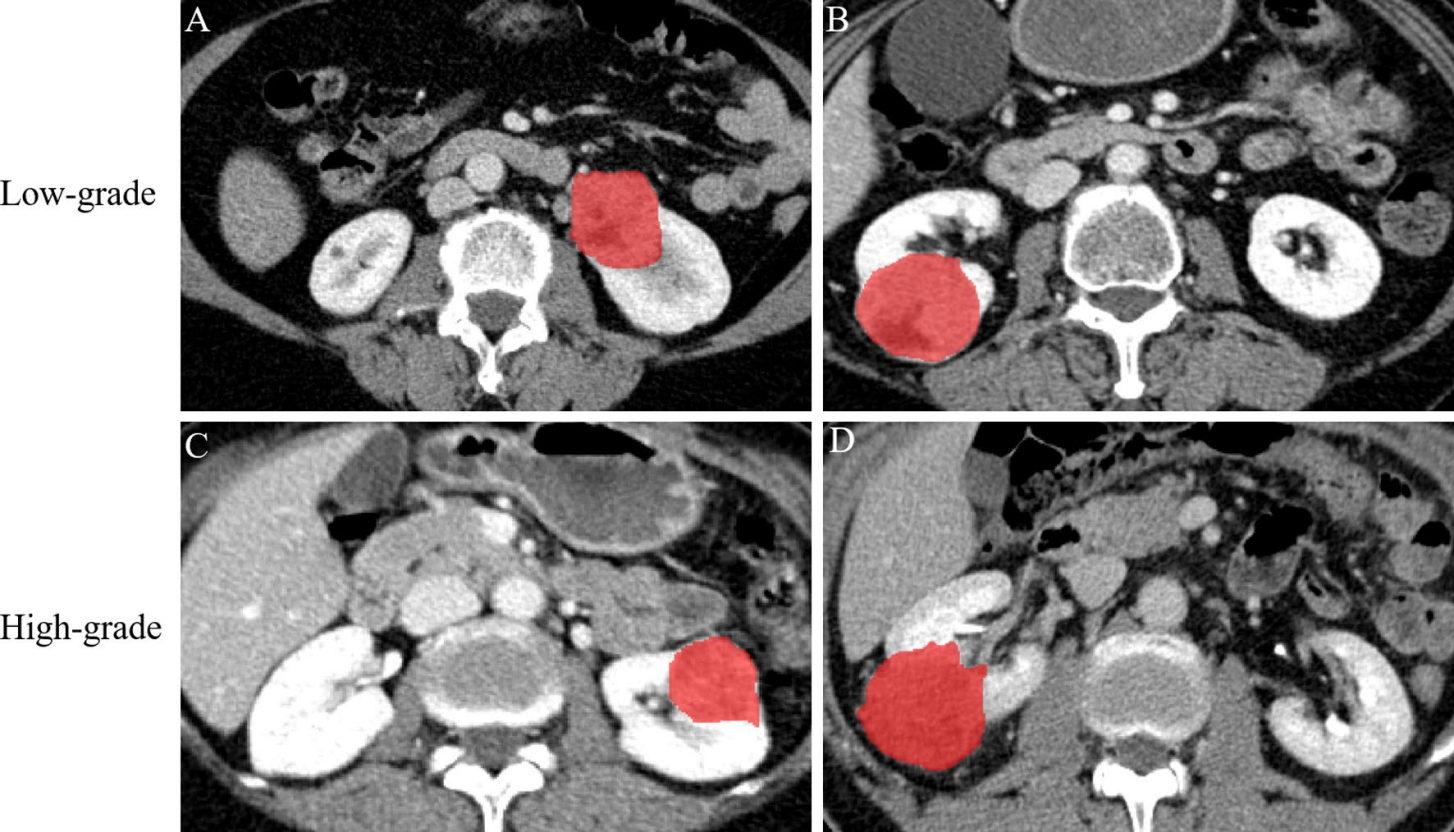
Examples of manually delineated regions of interest (ROIs) on NP images. **a, b** The delineation of ROI on two patients with low-grade CCRCC. **c, d** The delineation of ROI on two patients with high-grade CCRCC

All CT images were downloaded in DICOM format from the pictured archiving and communication system (Carestream, Canada) at their original dimensions and resolution and loaded into ITK-SNAP software version 3.8 [30]. A radiologist with ≥ 10 years of experience in abdominal imaging who was blinded to the pathological results (reader 1) meticulously manually delineated the regions of interest (ROIs) in a slice-by-slice manner (Fig. 2). To evaluate the reproducibility of radiomics features, ROI-based radiomics features of 30 randomly selected patients (from the whole study cohort) were re-extracted by reader 1 and another radiologist with 15 years of experience (reader 2). Thereafter, the intraclass correlation coefficient (ICC) values of both intra- and inter-observer agreement analyses were calculated to evaluate consistency and reproducibility in terms of feature extraction, where features with ICC values > 0.80 were included in the subsequent analysis. Inter-observer variation refers to the discrepancy between the results obtained by two or more observers performing the same ROI detection. Intra-observer variation refers to the discrepancy in the measurements of one observer when performing an experiment more than once.

### Radiomics feature extraction

All images were preprocessed before radiomics feature extraction as follows: first, the images and ROIs were resampled to an isotropic voxel size of 1 × 1 × 1 mm^3^ using B-spline interpolation; second, we focused on the chosen region and divided by standard deviation to normalize the images; third, the gray level of the image was discretized by a fixed bin width of 25 in the histogram. An open-source PyRadiomics library [31] was employed to extract radiomics features, which were divided into the following three subgroups: (1) descriptors of the size and shape of the ROI, such as the volume and maximum surface, compactness, and sphericity of the tumor; (2) first-order statistics features, such as the mean, median, maximum, and minimum values, that described the distribution of voxel intensities within tumor; and (3) second- and higher-order statistics features (texture features) that reflected changes in the gray levels of image space and were used to measure the inter-relationships between voxel distributions within tumor. Gray-level co-occurrence matrix, gray-level run-length matrix, gray-level size-zone matrix, and gray-level dependence matrix were included in these features.

### Prediction model construction

The following three models were built to predict the WHO/ISUP grade in this study: clinicoradiological, radiomics, and combined models. To construct the clinicoradiological model, univariate regression was first used to analyze radiological and clinical characteristics, such as sex, age, and intratumoral necrosis. Significant variables were further selected for the multivariate regression model. Finally, variables with *p *value < 0.05 were adopted. The radiomics-based ML model was constructed using a support vector machine (SVM). To obtain the top of prediction performance, different feature selection algorithms such as least absolute shrinkage and selection operator (LASSO), recursive feature elimination (RFE), and ReliefF were employed to select suitable radiomics features, and those features from different feature selection algorithms were fed into SVM for the prediction performance comparison, respectively. The combined model was constructed and analyzed using the SVM by gathering the selected radiological and clinical characteristics as well as radiomics features. All of these procedures were implemented using the scikit-learn library in Python (version 3.6).

### Model evaluation

Performance metrics, including sensitivity, specificity, accuracy, positive predictive value (PPV), negative predictive value (NPV), and area under the receiver operating characteristic (ROC) curve (AUC), were used to evaluate the performance of the three prediction models. The DeLong test was performed as a nonparametric approach for the comparison of ROC curves in AUC values. In the testing cohort, calibration curve analysis was used to assess the similarity between the predicted and observed outcomes of the model, accompanied by the Hosmer–Lemeshow test. Furthermore, decision curve analysis (DCA) was conducted to demonstrate the clinical net benefit of the model. The net reclassification index (NRI) was used to evaluate the prediction ability of the model in clinical utility. To minimize perturbation problems in feature selection and to examine the reproducibility of experimental results [32], we randomly assigned the patients to a training or testing cohort 10 times. Categorizing the original dataset into different cohorts was stratified and shuffled to ensure a similar CCRCC nuclear grade distribution across the datasets. Overall, 30% of the data were taken as an independent testing cohort, whereas the rest were taken as the training and validation cohorts for the model via fivefold cross-validation. Stratification into the training cohort was automatically performed without user intervention to avoid selection bias. Subsequently, the model was reconstructed and verified repeatedly.

### Statistical analysis

Categorical variables are expressed as counts (n) and percentages (%), whereas continuous variables are presented as mean values ± standard deviations or as medians with interquartile ranges. Differences in characteristics across the three datasets were analyzed using one-way analysis of variance or the Kruskal–Wallis test for normally or non-normally distributed continuous variables, followed by a post hoc test, as appropriate. Student’s t test or the Wilcoxon test was used for the comparison of continuous variables between groups. Categorical variables were subjected to the Chi-square test or Fisher’s exact test. The inter-observer agreement of CT findings for low- and high-grade CCRCCs between two radiologists was evaluated using kappa statistics. A forward stepwise regression was used to refine the regression model according to the Akaike information criterion. To correct for multiple comparisons, we adjusted the *p* values by false discovery rate correction using the Benjamini–Hochberg method [33]. All statistical analyses were performed using R software version 3.5.2 (http://www.rproject.org) with “pROC”, “rms”, and “DecisionCurve” packages. A two-tailed *p* value of < 0.05 was considered statistically significant.

## Results

### Clinicoradiological characteristics between the low- and high-grade groups

Out of 406 patients enrolled in this study, 240 were male and 166 were female, with an average age of 57.48 ± 12.10 years (range 16–83 years). The baseline clinical characteristics of patients are summarized in Table 1. In the patient cohort, 330 patients were diagnosed with low-grade CCRCC, whereas the rest were diagnosed with high-grade CCRCC. The majority of patients with high-grade tumors (*n* = 56, 73.7%) underwent radical nephrectomy, whereas most patients with low-grade tumors preferred partial nephrectomy (*n* = 192, 58.2%, *p* < 0.001). High-grade and low-grade cohorts significantly differed with respect to tumor size (5.82 ± 2.85 cm vs. 4.21 ± 2.02 cm; range: 0.8–14.6 cm, *p* < 0.001), hematuria symptoms (*p* = 0.023), distant metastasis (*p* = 0.035), intratumoral necrosis (*p* < 0.001), calcification (*p* < 0.001), violation of the renal capsule (*p* < 0.001), angiogenesis (*p* < 0.001), venous invasion (*p* < 0.001), and perinephric metastasis (*p* < 0.001).

**Table 1:** Clinical and radiological characteristics of patients involved in this research

Characteristics	Full cohort (*n* = 406)	Low grade (*n* = 330)	High grade (*n* = 76)	*t* value or *χ*^2^-value	*p *value
Age (years)	57.48 ± 12.10	57.12 ± 11.94	59.07 ± 12.73	1.268	0.205
*Gender, n (%)*					
Male	240 (59.1%)	189 (57.3%)	51 (67.1%)	2.471	0.116
Female	166 (40.9%)	141 (42.7%)	25 (32.9%)		
BMI (kg/m^2^)	24.30 ± 4.07	24.45 ± 4.04	23.69 ± 4.17	1.469	0.143
Smoking history, *n* (%)	135 (33.3%)	105 (31.8%)	30 (39.5%)	1.631	0.202
Hypertension, * n* (%)	147 (36.2%)	125 (37.9%)	22 (28.9%)	2.133	0.144
Diabetes, *n* (%)	62 (15.3%)	49 (14.8%)	13 (17.1%)	0.243	0.622
Tumor size (cm)	4.51 ± 2.29	4.21 ± 2.02	5.82 ± 2.85	4.674	< 0.001
*Tumor bearing, n (%*)					
Left	214 (52.7%)	178 (53.9%)	36 (47.4%)	1.070	0.301
Right	192 (47.3%)	152 (46.1%)	40 (52.6%)		
*Operative method, n (%)*					
Partial	213 (52.3%):	192 (58.2%):	20 (26.3%):	25.140	< 0.001
Radical	194 (47.7%)	138 (41.8%)	56 (73.7%)		
Hematuria, *n* (%)	49 (12.1%)	34 (10.3%)	15 (19.7%)	5.180	0.023
Flank pain, *n* (%)	63 (15.5%)	48 (14.5%)	15 (19.7%)	1.270	0.260
Distant Metastasis, *n* (%)	2 (0.5%)	0 (0.0%)	2 (2.6%)	–	0.035
Intratumoral necrosis, *n* (%)	192 (47.3%)	135 (40.9%)	57 (75.0%)	28.802	< 0.001
Cystic Degeneration, *n* (%)	39 (9.6%)	34 (10.3%)	5 (6.6%)	1.001	0.317
Calcification, *n* (%)	24 (5.9%)	12 (3.6%)	12 (15.8%)	14.292	< 0.001
Violation of the Renal Capsule, *n* (%)	64 (15.8%)	35 (10.6%)	29 (38.2%)	35.314	< 0.001
Angiogenesis, *n* (%)	250 (61.6%)	184 (55.8%)	66 (86.8%)	25.228	< 0.001
Venous invasion, *n* (%)	9 (2.2%)	2 (0.6%)	7 (9.2%)	17.316	< 0.001
Perinephric metastasis, *n* (%)	42 (10.3%)	18 (5.5%)	24 (31.6%)	45.456	< 0.001

*n* number, *BMI* body mass index

### Clinicoradiological characteristics among the training, validation, and testing cohorts

Table 2 shows the differences in clinicoradiological features between patients with low- and high-grade CCRCCs in the training, validation, and testing cohorts. Clinicoradiological characteristics of those are summarized in Table 3. Except for violation of the renal capsule (*P* < 0.001), no significant differences in either clinical or radiological features were identified between the different cohorts.

**Table 2:** Comparison of demographic and radiological characteristics between high- and low-grade CCRCCs

Characteristics	Training cohort				Validation cohort				Testing cohort			
	Low-grade	High-grade	*t *value or *χ*^2^-value	*p* value	Low-grade	High-grade	*t* value or * χ* ^2^-value	*p* value	Low-grade	High-grade	*t *value or *χ*^2^-value	*p* value
*Full cohort, n (%)*												
406 (100%)	184 (45.3%)	43 (10.6%)	–	–	46 (11.3%)	11 (2.7%)	–	–	100 (24.6%)	22 (5.4%)	–	–
Age (years)	56.87 ± 12.09	60.33 ± 11.13	2.93	0.088	55.63 ± 10.74	59.64 ± 11.17	1.22	0.28	58.25 ± 12.19	56.32 ± 16.14	0.40	0.53
*Gender, n (%)*												
Male	109 (59.2%)	27 (62.8%)	0.183	0.669	25 (54.3%)	7 (63.6%)	0.31	0.58	55 (55.0%)	17 (77.3%)	3.70	0.05
Female	75 (40.8%)	16 (37.2%)			21 (45.7%)	4 (36.4%)			45 (45.0%)	5 (22.7%)		
BMI (kg/m^2^)	24.25 ± 3.42	24.06 ± 5.00	0.087	0.768	24.18 ± 3.59	23.87 ± 2.19	0.08	0.78	24.93 ± 5.13	22.87 ± 2.96	3.30	0.07
Smoking history, n (%)	57 (31.0%)	18 (41.9%)	1.87	0.17	11 (23.9%)	4 (36.4%)	0.71	0.40	37 (37.0%)	8 (36.4%)	0.003	0.96
Hypertension, n (%)	67 (37.4%)	12 (27.9%)	1.11	0.29	17 (37.0%)	3 (27.3%)	0.37	0.55	41 (41.0%)	7 (31.8%)	0.64	0.43
Diabetes, n (%)	21 (11.4%)	9 (20.9%)	2.75	0.097	9 (19.6%)	0 (0.0%)	2.56	0.11	19 (19%)	4 (18.2%)	0.008	0.93
Tumor size (cm)	4.18 ± 1.86	5.76 ± 3.12	18.72	< 0.001	4.06 ± 2.37	6.23 ± 1.67	8.20	0.006	4.32 ± 2.15	5.75 ± 2.87	6.95	0.009
*Tumor location, n (%)*												
Left	103 (56.0%)	20 (46.5%)	1.26	0.26	21 (45.7%)	2 (20.0%)	2.23	0.135	54 (54.0%)	14 (63.6%)	0.68	0.41
Right	81 (44.0%)	23 (53.5%)			25 (54.3%)	8 (80.0%)			46 (46.0%)	8 (36.4%)		
*Operative method, n (%)*												
Partial	109 (59.2%)	11 (25.6%)	15.85	< 0.001	32 (69.6%)	2 (18.2)	9.74	0.002	51 (51.0%)	7 (31.8%)	2.66	0.10
Radical	75 (40.8%)	32 (74.4%)			14 (30.4%)	9 (81.8%)			49 (49.0%)	15 (68.2%)		
Hematuria, n (%)	13 (7.1%)	9 (20.9%)	7.66	0.006	10 (21.7%)	0 (0.0%)	2.90	0.089	11 (11.0%)	6 (27.3%)	3.98	0.046
Flank pain, n (%)	26 (14.1%)	6 (14.0%)	0.001	0.98	4 (8.7%)	3 (27.3%)	2.84	0.092	18 (18.0%)	6 (27.3%)	0.98	0.32
Distant Metastasis, n (%)	0 (0.0%)	1 (2.3%)	4.30	0.038	0 (0.0%)	1 (2.3%)	4.30	0.038	0 (0.0%)	1 (4.5%)	4.58	0.032
Necrosis, n (%)	76 (41.3%)	31 (72.1%)	13.26	< 0.001	19 (41.3%)	11 (100.0%)	12.67	< 0.001	40 (40.0%)	15 (68.2%)	5.79	0.016
Cystic Degeneration, n (%)	19 (10.4%)	4 (9.3%)	0.044	0.83	5 (10.9%)	1 (9.1%)	0.03	0.863	10 (10.0%)	0 (0.0%)	2.40	0.12
Calcification, n (%)	9 (4.9%)	6 (14.0%)	4.64	0.031	1 (2.2%)	2 (18.2%)	4.56	0.033	2 (2.0%)	4 (18.2%)	10.10	0.001
Violation of the Renal Capsule, n (%)	25 (13.6%)	19 (44.2%)	20.89	< 0.001	3 (6.5%)	0 (0.0%)	0.76	0.38	7 (7.0%)	10 (45.5%)	22.24	< 0.001
Angiogenesis, n (%)	101 (54.9%)	36 (83.7%)	12.11	0.001	23 (50.0%)	10 (90.9%)	6.10	0.014	60 (60.0%)	20 (90.9%)	7.63	0.006
Venous invasion, n (%)	1 (0.5%)	6 (14.0%)	20.97	< 0.001	0 (0.0%)	0 (0.0%)	–	–	1 (1.0%)	1 (4.5%)	1.41	0.24
Perinephric Metastasis, n (%)	12 (6.5%)	15 (34.9%)	26.75	< 0.001	2 (4.3%)	2 (18.2%)	2.60	0.11	4 (4.0%)	7 (31.8%)	17.01	< 0.001

**Table 3:** Clinical and radiological characteristics between three cohorts

Characteristics	Training cohort	Validation cohort	Testing cohort	*t *value or *χ*^2^-value	*p* value
*Full cohort, n (%)*					
406 (100%)	227 (55.9%)	57 (14.1%)	122 (30.0%)	–	–
*Clinical characteristics*					
Age (years)	57.52 ± 11.97	56.40 ± 10.84	57.90 ± 12.93	0.30	0.72
Gender, n (%)					
Male	136 (59.9%)	32 (56.1%)	72 (59.0%)	0.27	0.87
Female	91 (40.1%)	25 (43.9%)	50 (41.0%)		
BMI (kg/m^2^)	24.21 ± 3.75	24.12 ± 3.35	24.56 ± 4.87	0.35	0.71
Smoking history, n (%)	75 (33.0%)	15 (26.3%)	45 (36.9%)	1.97	0.37
Hypertension, n (%)	79 (34.8%)	20 (35.1%)	48 (39.3%)	0.75	0.69
Diabetes, n (%)	30 (13.2%)	9 (15.8%)	23 (18.9%)	1.96	0.38
Tumor location, n (%)					
Left	123 (54.2%)	24 (42.1%)	68 (55.7%)	3.69	0.16
Right	104 (45.8%)	33 (57.9%)	54 (44.3%)		
Operative method, n (%)					
Partial	120 (52.9%)	34 (59.6%)	58 (47.5%)	2.37	0.31
Radical	107 (47.1%)	23 (40.4%)	64 (52.5%)		
Hematuria, n (%)	22 (9.7%)	10 (17.5%)	17 (13.9%)	3.22	0.20
Flank pain, n (%)	32 (14.1%)	7 (12.3%)	24 (19.7%)	2.41	0.30
Distant Metastasis, n (%)	1 (0.4%)	0 (0.0%)	1 (0.8%)	0.56	0.76
*Imaging characteristics*					
Tumor size (cm)	4.48 ± 2.24	4.48 ± 2.40	4.58 ± 2.35	0.083	0.92
Necrosis, n (%)	107 (47.1%)	30 (52.6%)	55 (45.1%)	0.89	0.64
Cystic Degeneration, n (%)	23 (10.2%)	6 (10.5%)	10 (8.2%)	0.42	0.81
Calcification, n (%)	15 (6.6%)	3 (5.3%)	6 (4.9%)	0.46	0.80
Violation of the Renal Capsule, n (%)	44 (19.4%)	3 (5.3%) ^*^	17 (13.9%)	7.28	0.026
Angiogenesis, n (%)	137 (60.4%)	33 (57.9%)	80 (65.6%)	1.29	0.52
Venous invasion, n (%)	7 (3.1%)	0 (0.0%)	2 (1.6%)	2.27	0.32
Perinephric Metastasis, n (%)	27 (11.9%)	4 (7.0%)	11 (9.0%)	1.50	0.47

^*^ p < 0.05 for the comparison with patients in the training set

### Clinicoradiological model construction

Kappa analysis indicated that the inter-observer agreement of CT findings for low- and high-grade CCRCCs between the two radiologists were highly consistent, yielding kappa values of 0.779–0.923 (Table 4). Based on the results of univariate analysis, indicators such as tumor size, hematuria symptoms, intratumoral necrosis, calcification, violation of the renal capsule, angiogenesis, venous invasion, and perinephric metastasis showed significantly different between high- and low-grade groups were included in the multivariate analysis (Table 4). As a result, tumor necrosis (odds ratio [OR] = 2.745, 95% confidence interval [CI] 1.424–5.292, *p* = 0.003), tumor calcification (OR = 4.293, 95% CI 1.629–11.314, *p* = 0.003), and angiogenesis (OR = 3.805, 95% CI 1.741–8.313, *p* = 0.001) were presumed to be independent factors of high-grade level and thus acted as clinical features in the clinicoradiological model construction.

**Table 4:** Risk factors for WHO/ISUP nuclear grade of CCRCC according to univariate and multivariate analysis

Characteristics	Kappa value	Univariate analysis			Multivariate analysis		
		OR	95% CI	*p *value	OR	95% CI	*p *value
Tumor size	0.875	7.845	3.652–12.355	< 0.001	0.992	0.852–1.155	0.918
Hematuria	0.867	2.106	11.082–4.101	0.026	1.145	0.482–2.720	0.758
Distant metastasis	0.831	–	–	-	–	–	0.999
Intratumoral necrosis	0.854	4.465	2.543–7.838	< 0.001	2.745	1.424–5.292	0.003
Calcification	0.905	4.502	1.965–10.311	< 0.001	4.293	1.629–11.314	0.003
Violation of renal capsule	0.814	5.092	2.854–9.086	< 0.001	1.642	1.642–3.446	0.190
Angiogenesis	0.779	11.486	5.766–22.879	< 0.001	3.805	1.741–8.313	0.001
Venous invasion	0.923	16.400	3.336–80.624	< 0.001	4.037	0.663–24.583	0.130
Perinephric metastasis	0.867	7.849	3.989–15.446	< 0.001	3.032	1.283–7.168	0.011

### Radiomics feature extraction and radiomics model construction

**Fig. 3:**
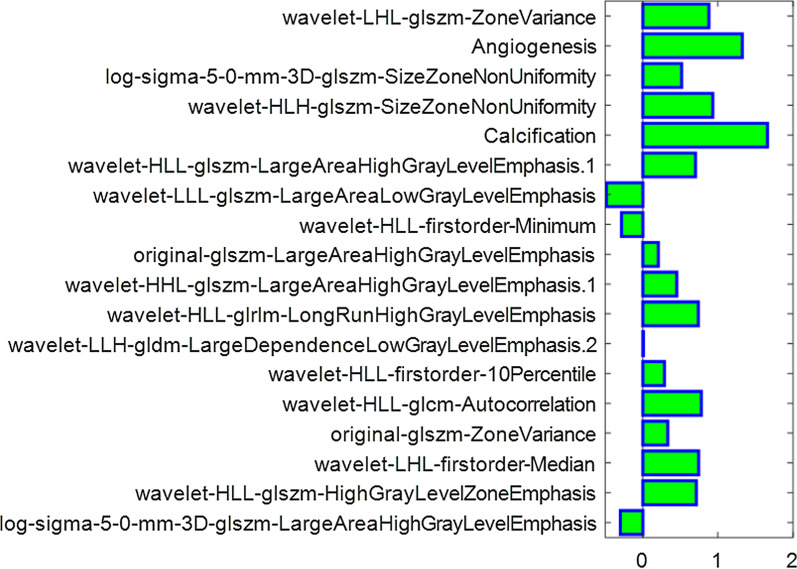
Diagram of the feature selection result. The bar plot represents the weight of each feature in the support vector machine

**Fig. 4:**
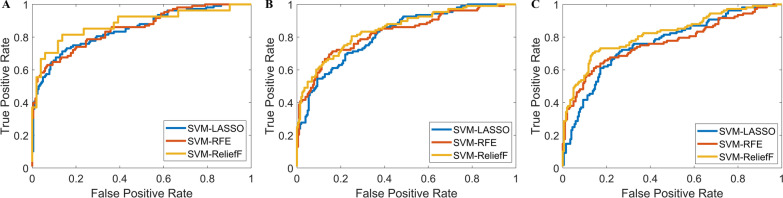
Predictive performance of three machine learning algorithms: SVM-LASSO, SVM-RFE, and SVM-ReliefF. **a** Receiver operating characteristic (ROC) curve analysis for the training cohort. **b** ROC curve analysis for the validation cohort. **c** ROC curve analysis for the testing cohort

A total of 972 features of NP CT images were extracted from the ROIs using the PyRadiomics package, and those with ICC values > 0.8 on both intra- and inter-observer agreement analyses were retained. A dimensionality reduction was conducted, and 16 features were finally selected to build the radiomics model. The selected features are shown in Fig. 3. The classifiers such as SVM-LASSO, SVM-RFE, and SVM-ReliefF were utilized  for model construction. Their ability to distinguish low- from high-grade CCRCCs is summarized in Table 5. In testing cohort, SVM-ReliefF yielded an AUC value of 0.787 (95% CI 0.710–0.892), whereas the AUC values of SVM-RFE and SVM-LASSO were 0.761 (95% CI 0.648–0.893) and 0.754 (95% CI 0.652–0.889). With an accuracy of 0.734 (95% CI 0.616–0.827), a sensitivity of 0.822 (95% CI 0.737–0.919), and a specificity of 0.765 (95% CI 0.634–892), SVM-ReliefF turned into the best performer among the three classifiers. A comparison of the AUCs of the three algorithms in each data set is displayed in Fig. 4.

**Table 5:** Predictive performance of three classifiers: SVM-LASSO, SVM-RFE, and SVM-ReliefF

Classifier	SVM-LASSO			SVM-RFE			SVM-ReliefF		
	Training cohort	Validation cohort	Testing cohort	Training cohort	Validation cohort	Testing cohort	Training cohort	Validation cohort	Testing cohort
AUC	0.838 [0.721–0.934]	0.795 [0.674–0.899]	0.754 [0.652–0.889]	0.842 [0.747–0.945]	0.803 [0.687–0.904]	0.761 [0.648–0.893]	0.860 [0.759–0.963]	0.824 [0.736–0.915]	0.787 [0.710–0.892]
Accuracy (%)	76.70 [68.05–85.26]	73.94 [73.49–89.75]	68.78 [55.67–82.89]	77.36 [65.35–86.25]	74.11 [62.98–87.19]	71.58 [59.87–83.74]	83.61 [75.85–92.65]	77.14 [69.38–86.05]	73.42 [61.63–82.66]
Sensitivity (%)	77.57 [64.45–87.31]	73.48 [60.67–86.48]	70.07 [61.71–82.99]	80.33 [69.35–89.79]	75.47 [63.78–89.36]	71.25 [61.36–80.74]	84.78 [74.36–93.74]	78.72 [64.35–91.48]	82.15 [73.74–91.92]
Specificity (%)	81.22 [72.19–89.24]	79.63 [61.34–70.49]	80.30 [71.58–89.43]	84.31 [73.74–92.18]	83.04 [71.39–95.46]	79.22 [66.57–88.34]	82.67 [74.35–93.14]	80.33 [69.74–92.26]	76.48 [63.36–89.17]
PPV (%)	76.88 [62.38–85.69]	73.86 [59.67–88.74]	69.71 [60.98–78.35]	79.46 [64.38–91.59]	74.78 [62.88–86.74]	72.98 [67.64–83.35]	77.79 [69.16–85.97]	75.98 [68.30–83.57]	74.82 [65.87–83.64]
NPV (%)	84.12 [76.41–95.37]	80.48 [71.70–92.26]	79.25 [66.28–91.33]	85.34 [75.65–96.43]	83.92 [74.54–91.35]	80.25 [72.36–92.17]	83.90 [72.74–95.46]	80.43 [71.32–89.91]	82.62 [71.64–92.35]

Data in parentheses are 95% confidence intervals

*PPV* positive predictive value, *NPV* negative predictive value

### Comparison of the performance among clinicoradiological, radiomics, and combined models

**Fig. 5:**
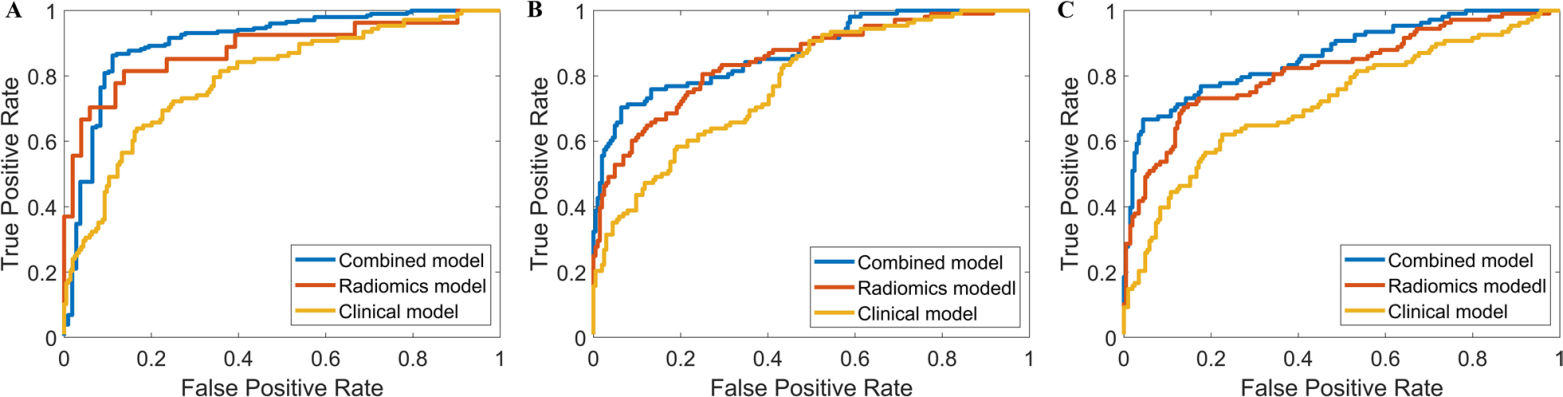
Predictive performance of SVM-ReliefF classifier in clinicoradiological, radiomics, and combined models. **a** Receiver operating characteristic (ROC) curve analysis for the training cohort. **b **ROC curve analysis for the validation cohort. **c** ROC curve analysis for the testing cohort

As the optimum algorithm of the three classifiers, SVM-ReliefF was chosen to predict WHO/ISUP nuclear grade of CCRCC by analyzing features contained in clinicoradiological, radiomics, and combined models. AUC, sensitivity, specificity, PPV, and NPV were calculated to assess the prediction performance of models. As exhibited in Fig. 5a–c, compared with the clinicoradiological and radiomics models, the combined model showed the best predictive efficacy in distinguishing low- from high-grade CCRCCs with the highest AUC values in training, validation, and testing cohorts (*p* < 0.05, DeLong test). The AUC values of the combined model were 0.887 (95% CI 0.798–0.952) and 0.859 (95% CI 0.748–0.935) in the training and validation cohorts, which were higher than those of the radiomics model with AUC values of 0.860 (95% CI 0.759–0.963) and 0.824 (95% CI 0.736–0.915), while the clinicoradiological model demonstrated the worst performance with AUC values of 0.752 (95% CI 0.649–0.870) and 0.703 (95% CI 0.592–0.844) respectively. In the testing cohort, the combined model yielded an AUC value of 0.828 (95% CI 0.731–0.929) (radiomics model: 0.787 [95% CI 0.710–0.892]; clinicoradiological model: 0.637 [95% CI 0.511–0.769]), with an accuracy of 0.816 (95% CI 0.742–0.925), a sensitivity of 0.856 (95% CI 0.778–0.916) and a specificity of 0.780 (95% CI 0.695–0.857), which showed the best prediction performance in differentiating the WHO/ISUP nuclear grade. The detailed predictive performance of the three models are summarized in Table 6, and the confusion matrices of the combined model in the testing cohort for the random splitting process of 10-times runs are shown in Additional file 1: Figure S2.

**Table 6:** Predictive performance of combined model, radiomics model, and clinicoradiological model

Model	Combined model			Radiomics model			Clinicoradiological model		
	Training cohort	Validation cohort	Testing cohort	Training cohort	Validation cohort	Testing cohort	Training cohort	Validation cohort	Testing cohort
AUC	0.887 [0.798–0.952]	0.859 [0.748–0.935]	0.828 [0.731–0.929]	0.860 [0.759–0.963]	0.824 [0.736–0.915]	0.787 [0.710–0.892]	0.752 [0.649–0.870]	0.703 [0.592–0.844]	0.637 [0.511–0.769]
Accuracy (%)	85.24 [76.75–90.14]	82.76 [75.99–91.36]	81.62 [74.18–92.45]	83.61 [75.85–92.65]	77.14 [69.38–86.05]	73.42 [61.63–82.66]	69.33 [56.54–83.76]	62.87 [51.74–75.36]	56.35 [42.48–69.49]
Sensitivity (%)	89.77 [80.63–96.98]	84.94 [76.72–92.49]	85.56 [77.82–91.58]	84.78 [74.36–93.74]	78.72 [64.35–91.48]	82.15 [73.74–91.92]	71.86 [59.43–83.82]	73.89 [65.95–83.47]	65.78 [46.89–80.87]
Specificity (%)	84.47 [72.60–91.65]	83.42 [74.32–92.18]	78.01 [69.45–85.74]	82.67 [74.35–93.14]	80.33 [69.74–92.26]	76.48 [63.36–89.17]	70.42 [61.36–83.47]	66.86 [56.63–78.10]	64.23 [52.36–80.62]
PPV (%)	82.34 [73.87–91.59]	81.52 [73.67–87.59]	78.36 [70.16–86.47]	77.79 [69.16–85.97]	75.98 [68.30–83.57]	74.82 [65.87–83.64]	68.24 [56.25–81.39]	65.71 [52.63–78.78]	60.99 [48.34–73.76]
NPV (%)	87.65 [79.48–97.86]	86.77 [78.43–91.17]	85.60 [79.98–90.35]	83.90 [72.74–95.46]	80.43 [71.32–89.91]	82.62 [71.64–92.35]	74.22 [65.61–86.34]	70.98 [61.05–82.77]	73.17 [60.05–87.47]

Data in parentheses are 95% CIs

### Clinical usefulness

**Fig. 6:**
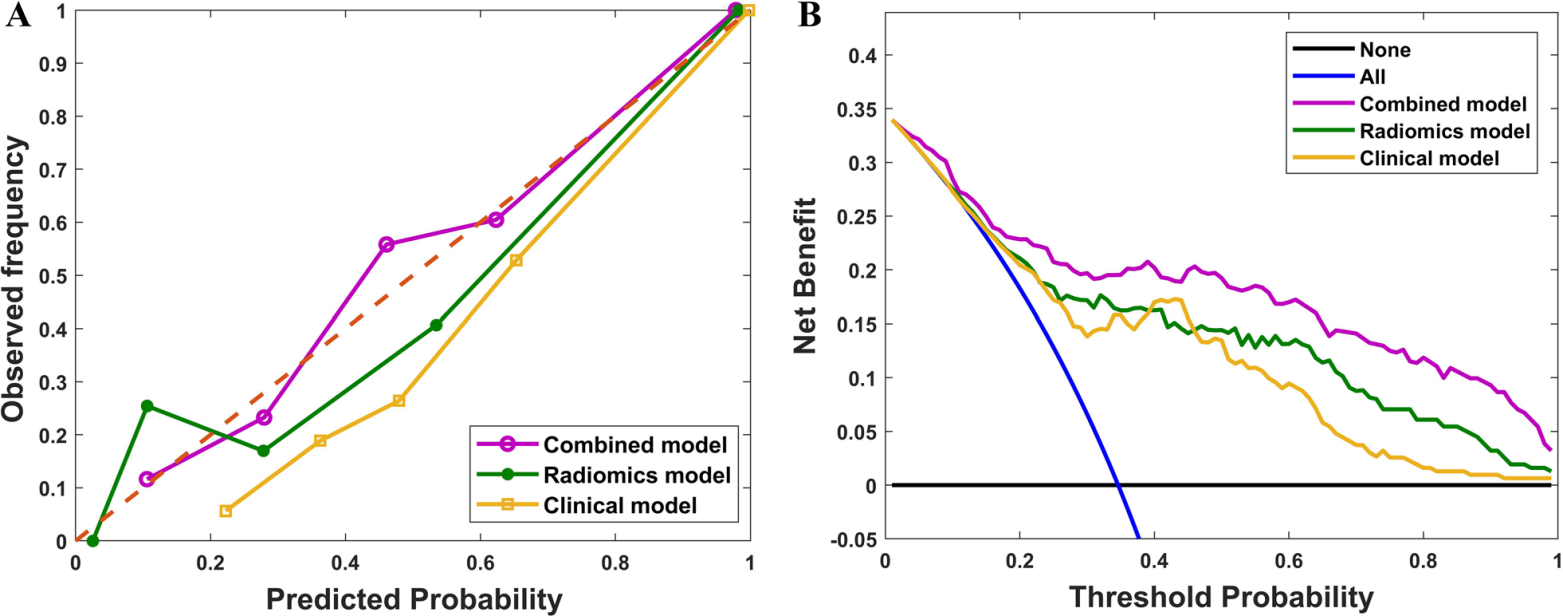
**a** Calibration curve and (**b**) decision curve analysis of the combined model, radiomics model, and clinicoradiological model in the testing cohort

The calibration curves of these three models for predicting low- and high-nuclear grade in CCRCC are shown in Fig. 6a. The calibration curve for the combined model demonstrated good agreement between observations and predictions in the testing cohort, accompanied by the Hosmer–Lemeshow test (*p* = 0.487, Fig. 6a) and followed by the radiomics model (*p* = 0.321, Fig. 6a). However, there were differences between observations and predictions for the clinicoradiological model in the testing cohort (*p* = 0.04, Fig. 6a). DCA indicated a higher net benefit for the combined model in distinguishing low- from high-grade CCRCCs than the other models (Fig. 6b). The threshold probability was within the range of 0.15–0.98. In the testing cohort, both the combined and radiomics models achieved better discrimination performance than the clinicoradiological model (*p* = 0.010 and 0.021, NRI test). Additionally, the discrimination ability of the combined model was also superior to that of the radiomics model (*p* = 0.038, NRI test).

## Discussion

In this study, we utilized NP CT based radiomics features combined with clinicoradiological characteristics to build three models such as the clinicoradiological, radiomics and combined models for distinguishing between low-and high-grade CCRCCs. The results demonstrated that NP CT based radiomics was valuable in predicting the WHO/ISUP nuclear grade of CCRCC, and associating the radiomics features with clinicoradiological characteristics could improve the predictive performance, compared with clinicoradiological and radiomics models alone. The combined model exhibited the best predictive performance and clinical usefulness with satisfactory reproducibility and reliability.

Although percutaneous biopsy is the routine way to identify the preoperative pathology grade, it is an invasive approach, and patients may suffer from sampling bias and the risk of complications [12, 13]. Some emerging imaging technologies such as dual-energy spectral CT, intravoxel incoherent motion imaging and diffusion kurtosis imaging could provide valuable information on the assessment of pathological grading of CCRCC [34, 35]. As a recommended noninvasive detection technology for CCRCC, CT may provide to improve the accuracy of percutaneous biopsy. CT radiomics as a burgeoning technique, is able to quantify tumor heterogeneity by the spatial arrangement of imaging voxels with signal intensity variations and detect the imperceptible differences of the intensity distribution in medical images, thus noninvasively predicting pathological grade of tumor with outstanding performance [36–38]. Recently, the WHO/ISUP grading system has taken the place of the former Fuhrman grading system and received acceptance in current clinical practice [39]. There are only a few published papers that have studied the application of CT radiomics to predicting the WHO/ISUP nuclear grade of CCRCC [40–43]. However, no previous studies used radiomics features extracted from NP CT images combined with clinicoradiological characteristics to develop the prediction model.

Most previous studies constructed ML models only based on CT radiomics features, which ignored the importance of traditional clinical and radiological information [26, 41, 44]. In our study, some parameters with clinical and radiological information that have the potential to be risk factors in the WHO/ISUP nuclear grade of CCRCC determined by multivariate regression model were fed into ML model, and the radiomics features combined with the clinicoradiological characteristics showed a better performance for the discrimination of CCRCC grades. Our result is in concordance with the results of previous studies [22, 40, 42, 45–47], and this is reinforced by the results of previous studies on the association between clinicoradiological characteristics and the nuclear grade of CCRCC [22, 48]. Xu et al. [49] observed that coagulative necrosis often occurs in the CT images of patients with high-grade CCRCC. In addition, our study also found intratumoral necrosis, calcification, angiogenesis, and perinephric metastasis could be risk factors of the pathological grading of CCRCC. The previously mentioned studies have shown the potential of quantitative CT features in preoperatively predicting the WHO/ISUP nuclear grade of CCRCC, but their sample sizes were relatively small. Our study with a larger sample size would provide support for verification of the reproducibility of CT radiomics in the application of predicting WHO/ISUP nuclear grade of CCRCC using an independent testing cohort. Furthermore, we firstly demonstrated that the radiomics features from only NP CT images could obtain a preferable predictive performance in distinguishing low- from high-grade CCRCCs.

The preoperative noninvasive knowledge of CCRCC grades may contribute the clinical managements and impact clinical decisions. The new WHO/ISUP grading system is a prognostic factor for CCRCC whose grades were strongly related to patient outcomes and tumor biological behavior [50]. If low-grade CCRCC can be identified preoperatively, the treatment may be different, and the patients with low-grade CCRCC may be candidates for less invasive procedures, such as radiofrequency ablation and nephron-saving surgery, whereas radical interventions are strongly recommended in patients with high-grade CCRCC [11]. Moreover, partial nephrectomy can preserve partial renal function, thus reducing rates of infection, overall mortality and the incidence of cardiovascular disease [51]. In the clinical management, patients with low-grade CCRCC are less likely to suffer from paraneoplastic syndrome and distant metastasis, so accurately preoperative prediction of CCRCC grades may reduce unnecessary examinations, such as positron emission tomography-computed tomography and radionuclide imaging, decreasing the economic burden and incidence of complications resulting from the usage of contrast agent. Considering the latest update of the European Association of Urology Guidelines on renal cell carcinoma [7], patients with suspicious CCRCC are strongly recommended to use multiphasic contrast-enhanced CT imaging of the abdomen for diagnostic assessment and staging of renal tumors. Therefore, medical images can become a valuable source of information, and radiomics may be used as a noninvasive method for characterizing and classifying lesions. Compared with percutaneous biopsy, the radiomics has the advantages of noninvasion, easy-to-repeat operation and no complications. Our result indicates that combining NP CT based radiomics and clinicoradiological characteristics would provide good predictive performance in distinguishing between patients with low- and high-grade CCRCCs. This could provide a reference for clinicians to choose a suitable treatment strategy. However, further larger prospective or prospective studies with multi-centric data are necessary to validate the performance of our proposed combined model in the future. A good performance does not always imply a clinically applicable and reliable model [52], and however, we found that most previous studies did not evaluate the clinical utility of their models [21, 22]. In our study, we used calibration curve and decision curve analyses to evaluate the discrimination performance of the three predictive models, which showed the combined model has higher clinical usefulness with a good agreement between observations and predictions and a preferable discrimination performance, thus indicating practical value.

This study has several limitations. First, although 406 subjects with all-sided data were included, this retrospective study was conducted in a single institution, which may inevitably result in selection bias and make it less generalizable to other institutions. Therefore, further studies should enroll the larger simple sizes from different centers and scanners to improve the generalization of the prediction model. Moreover, only single-phase CT images were used in this study, and comparison with other phases should be considered. Second, an automatic segmentation algorithm should be developed to replace the manually sketching of ROI to increase the stability of prediction model. Third, although we have performed calibration statistics and decision curve analyses on the prediction models and revealed that the combined model had the best discrimination ability, the clinical application should be further validated using larger prospective or prospective studies with multi-centric data. Fourth, CCRCC is a subtype of malignant renal tumor. Despite its high occurrence, other renal cancer subtypes could have similar radiological features, and therefore, should be evaluated in future studies.

In conclusion, we demonstrated that NP CT images could become a valuable source of information, and radiomics analysis of those may be used as a potentially noninvasive method for distinguishing low- from high-grade CCRCCs. The ML model associating the radiomics features with clinicoradiological characteristics could improve the predictive performance for WHO/ISUP nuclear grade of CCRCC, which may be a promising and feasible way to assist in the clinical managements and therapeutic decisions.

## Supplementary Information

**Additional file 1**. Supplementary figures.

## Data Availability

The original contributions presented in the study are included in the article.

## Abbreviations

AUC: Area under the ROC curve
BMI: Body mass index
CCRCC: Clear cell renal cell carcinoma
CMP: Corticomedullary phase
CT: Computed tomography
DCA: Decision curve analysis
FGS: Fuhrman grading system
ICC: Intraclass correlation coefficient
LASSO: Least absolute shrinkage and selection operator
ML: Machine learning
NP: Nephrographic phase
NPV: Negative predictive value
NRI: Net reclassification index
PACS: Pictured archiving and communication system
PPV: Positive predictive value
RFE: Recursive feature elimination
ROC: Receiver operating characteristic
ROI: Region of interest
SVM: Support vector machine
WHO/ISUP: World Health Organization/International Society of Urological Pathology
