# Supplementary material for: Machine learning-based CT radiomics approach for predicting WHO/ISUP nuclear grade of clear cell renal cell carcinoma: an exploratory and comparative study
Source: Insights Imaging. 2021 Nov 20;12:170. doi: 10.1186/s13244-021-01107-1 (PMC8605949; doi:10.1186/s13244-021-01107-1)
Supplement: Supplementary file 1 — Additional file 1. Supplementary figures. [file 13244_2021_1107_MOESM1_ESM.docx]

**ELECTRONIC SUPPLEMENTARY MATERIAL**


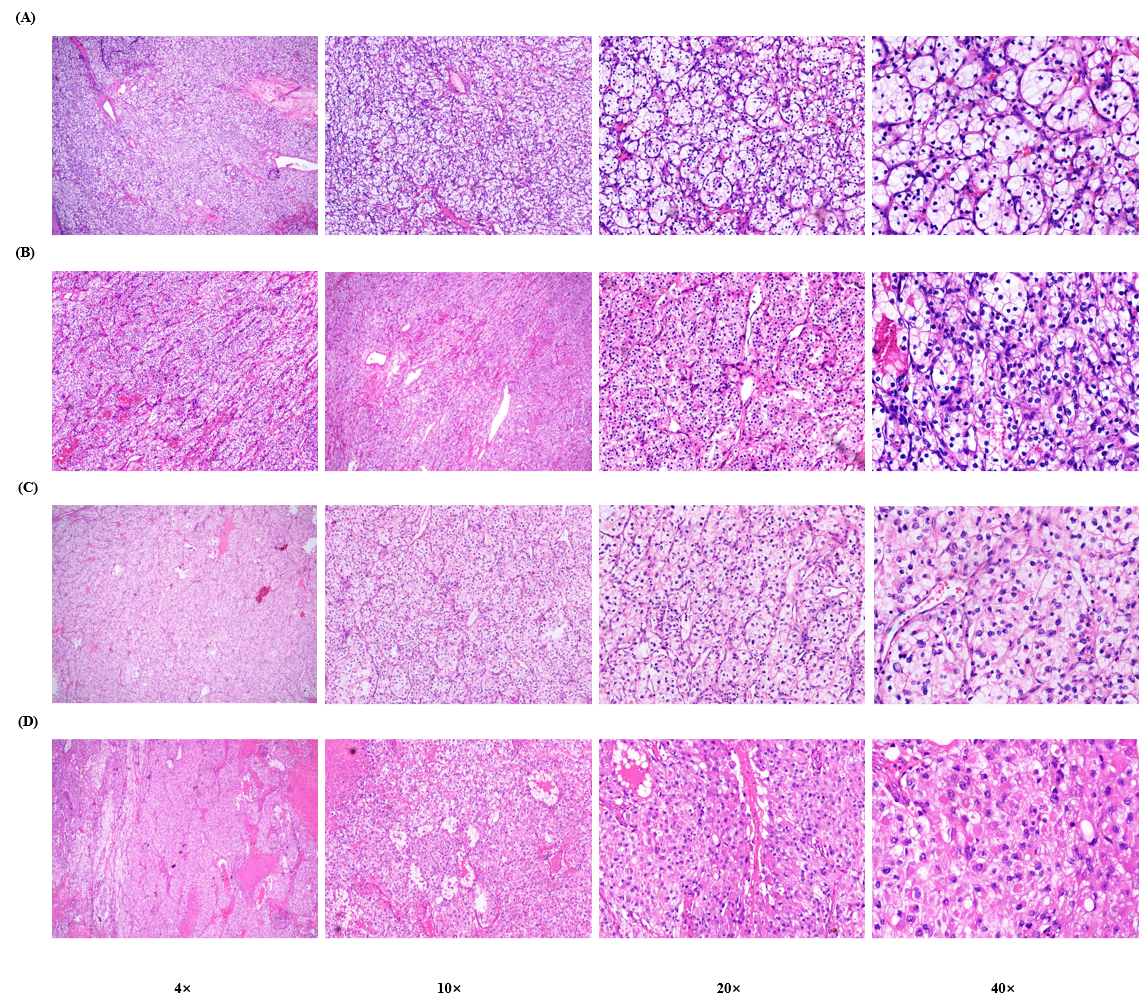


**Figure S1.** Typical hematoxylin-eosin staining slides with different magnifications (4×,10×,20×,40×) from four patients with WHO/ISUP grade I-IV. (A) Grade I. (B) Grade II. (C) Grade III. (D) Grade IV.


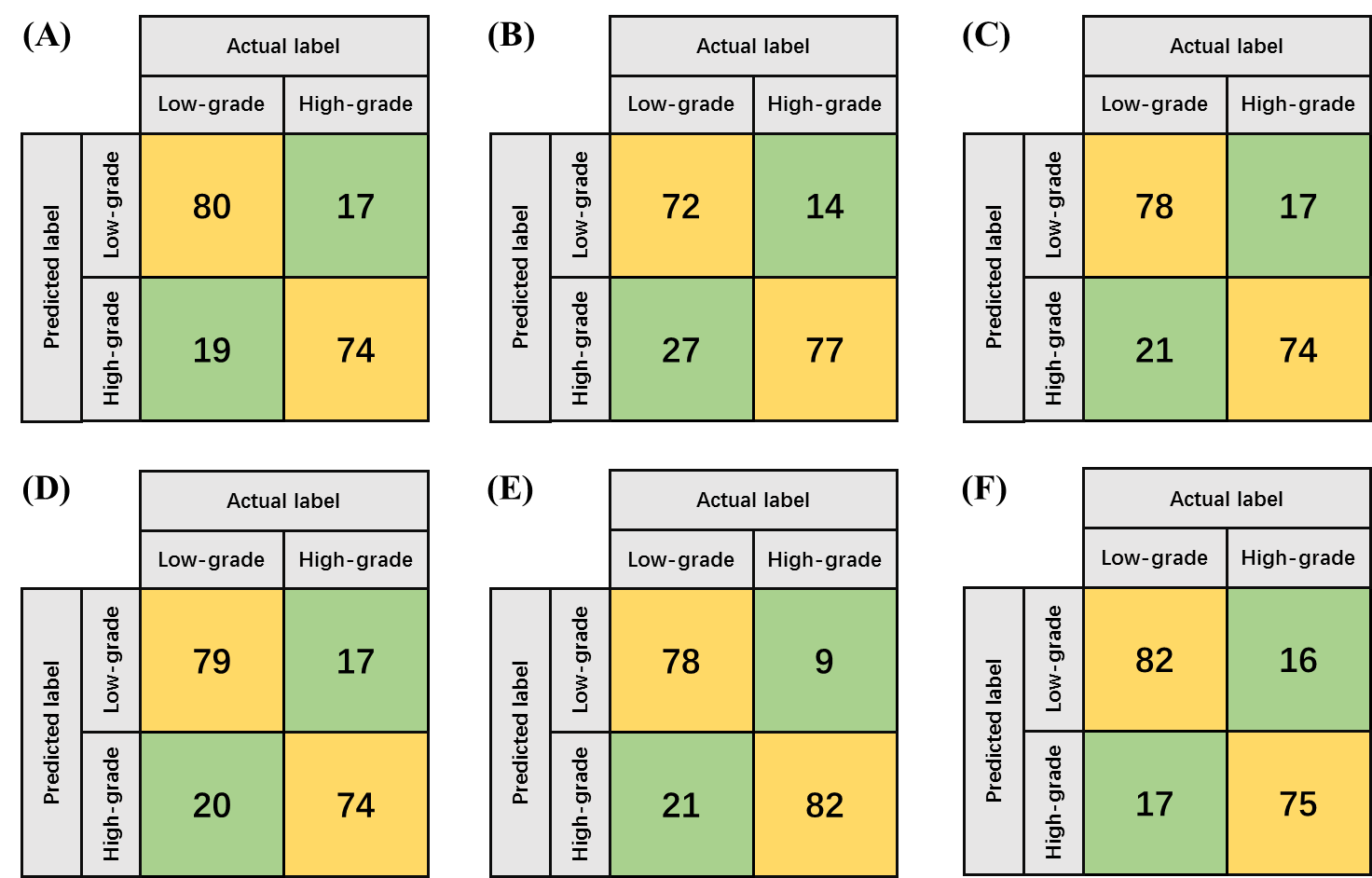


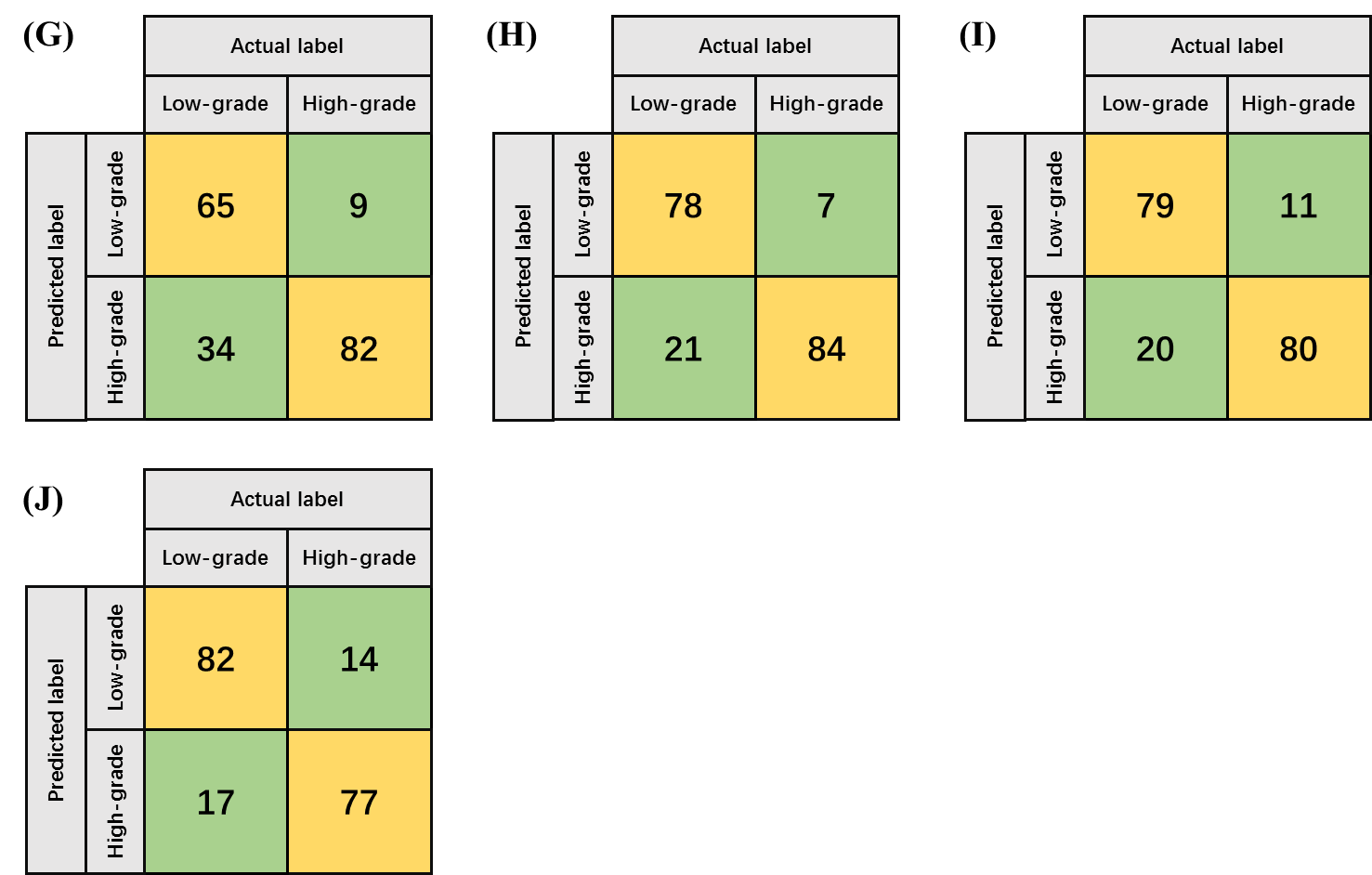


**Figure S2.** The Confusion Matrices of combined model in testing cohorts for the random splitting process of 10-times runs.
